# Anisotropic connectivity implements motion-based prediction in a spiking neural network

**DOI:** 10.3389/fncom.2013.00112

**Published:** 2013-09-17

**Authors:** Bernhard A. Kaplan, Anders Lansner, Guillaume S. Masson, Laurent U. Perrinet

**Affiliations:** ^1^Department of Computational Biology, Royal Institute of TechnologyStockholm, Sweden; ^2^Stockholm Brain Institute, Karolinska InstituteStockholm, Sweden; ^3^Department of Numerical Analysis and Computer Science, Stockholm UniversityStockholm, Sweden; ^4^Institut de Neurosciences de la Timone, UMR7289, Centre National de la Recherche Scientifique & Aix-Marseille UniversitéMarseille, France

**Keywords:** motion detection, motion extrapolation, probabilistic representation, predictive coding, network of spiking neurons, large-scale neuromorphic systems

## Abstract

Predictive coding hypothesizes that the brain explicitly infers upcoming sensory input to establish a coherent representation of the world. Although it is becoming generally accepted, it is not clear on which level spiking neural networks may implement predictive coding and what function their connectivity may have. We present a network model of conductance-based integrate-and-fire neurons inspired by the architecture of retinotopic cortical areas that assumes predictive coding is implemented through network connectivity, namely in the connection delays and in selectiveness for the tuning properties of source and target cells. We show that the applied connection pattern leads to motion-based prediction in an experiment tracking a moving dot. In contrast to our proposed model, a network with random or isotropic connectivity fails to predict the path when the moving dot disappears. Furthermore, we show that a simple linear decoding approach is sufficient to transform neuronal spiking activity into a probabilistic estimate for reading out the target trajectory.

## 1. Introduction

### 1.1. Problem statement

**Figure 1:**
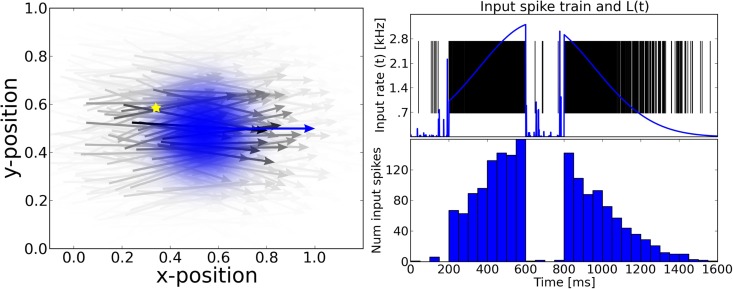
**The motion extrapolation problem**. Sensory input, such as the smooth motion of a dot in visual space, may be perturbed by disruption of sensory drive, like when the eye blinks during a visual stimulation. It is essential that some mechanisms may fill this blank: this defines the motion extrapolation problem. We first define the problem by parameterizing a generic input and its perturbation. **Left:** The input is a Gaussian hill of activity in a topographically organized space, moving on a straight trajectory. We show here a snapshot in time of the input (blue) and the resulting input activity to the network (gray) for a period of 400 ms. This corresponds for instance to the activation of a low-level visual area to a single dot represented by a bell-shaped hill of activity (blue blurred circle). In addition, this input carries information about the motion of the object (blue arrow) and drives neurons which have a close selectivity in position and velocity (gray arrows). **Right:** We show the time course of the input for one representative neuron (denoted by the yellow star in the left panel). **Top:** The blue trace shows the envelope of the inhomogeneous Poisson process that creates the input spike train. For 0 ms < *t* ≤ 200 ms and 600 ms < *t* ≤ 800 ms the stimulus is blanked, that is, that all neurons in the sensory layer receive input from a Poisson process with the same rate. We permuted the input vector fed into the network among all the cells in the network for each time step during the blank. Black vertical lines indicate input spikes. **Bottom:** Histogram of the input spike train with a bin size of 50 ms. This shows clearly the missing information during the blank. We define the goal of solving the motion extrapolation problem as representing the prediction of information on motion (speed and position) during the blank.

In a dynamical world, prediction is a highly relevant evolutionary advantage. This is crucial in sensory systems, as the raw data that is processed is most often noisy, and possibly ambiguous or distorted. Take for example the task performed by the primate visual system of tracking the trajectory of a moving object and accurately moving the eyes in order to stabilize the image on the retina. The image of the object may be blurred, or the measure of its velocity may depend on its geometry instead of its trajectory. Another problem occurs when the object is occluded, or simply when the observer blinks. It is an advantage to be able to predict the position and speed of the object at the end of this blanking period. This problem is classically referred to as *motion extrapolation* (see Figure 1). While predictive coding mechanisms may have different aspects and occur at different levels ranging from the retina to higher level areas (Gollisch and Meister, 2010), we will focus on this particular phenomenon as prototypical example.

Particularly in primates, object motion information is extracted along a cascade of feed-forward cortical areas, where primary visual area (V1) extracts local motion information that is integrated in extra-striate middle temporal (MT) and medial superior temporal (MST) areas (Newsome et al., 1988). MT and MST process visual motion and oculomotor signals driving pursuit (see Ilg, 1997 for a review) and are therefore key elements in motion extrapolation. Specifically, we will focus on the dynamics of neural activity during the period without informative sensory input (to which we will refer as the blank) and just after its reappearance. Indeed, the capacity of the dynamics to transform such fragmented input into a correct, continuous representation is a major pressure on the evolution of the visual system (Gollisch and Meister, 2010). It was shown in the monkey visual system that neural activity was mostly absent during the blank in lower areas of the visual hierarchy while it was maintained in some higher level areas (Assad and Maunsell, 1995). More precisely, neural activity in MT is driven by the motion of the dot and quickly devolves to spontaneous activity during a blank, while activity in its efferent area MST is maintained to the level of neural activity expected if the dot was not blanked when there is no retinal image motion. This can happen during a transient image occlusion (Newsome et al., 1988) or while tracking an imaginary target covering the visual field outside of the receptive field currently being recorded (Ilg and Thier, 2003). Similar sustained activity during target occlusion has been found in primate posterior parietal cortex, and it is linked to image motion prior to target disappearance (Assad and Maunsell, 1995), that is, by a predictive signal.

Motion extrapolation is also seen in lower level neuronal structures, such as the retina (Berry et al., 1999), and calls for a more generic computational framework. However, direct evidence for such neural mechanisms is still lacking. Before proposing a solution using a connectivity pattern based on motion-based prediction, we will first review some existing experimental and theoretical evidence. Along this study, our aim is to provide a basis for future applications of neuromorphic hardware (Schemmel et al., 2010; Brüderle et al., 2011).

### 1.2. Neuro-physiological correlates of prediction for motion extrapolation

At the neural level, it seems that the topography of neural representation is an essential constraint to prediction. Indeed, it is more efficient that populations of neurons that represent similar parameters should be adjacent. This is due to the cost of wiring neurons (length and volume of axon and dendrites) Chklovskii et al. (2002) but also due to the limited speed of information propagation in neural wires. Such aspect is particularly acute on the surface of the cerebral cortex and this hypothesis has been an efficient construct to understand the organization of visual areas (Miikkulainen et al., 2005). This is also implemented in other cerebral structures and species such as the convergence of inputs from place cells in the hippocampus of rats that code for path integration of body position in an environment (McNaughton et al., 2006). Physiological evidence shows that similar mechanisms are present in the deep superior colliculus of primates allowing for the integration of the belief on the position of a visual target in visual space for the guidance of saccadic or smooth eye movements (Krauzlis, 2004). Here, we will focus on low-level visual areas based on the neurophysiology of the macaque brain (V1, MT and MST), but we will keep a rather generic formulation to explore the functional role of some key parameters.

Neurons in such areas receive connections from neighboring neurons in the same cortical area (local connectivity) but also respectively by feed-forward or feed-back connections from lower or higher areas. Focusing on area MT, early physiological studies in macaque monkey identified this area as a specialized module for visual motion processing (Dubner and Zeki, 1971; Allman et al., 1973). This involves extracting speed and direction of the moving object. MT neurons respond selectively to visual motion and are tuned for local speed and direction of luminance features moving in their receptive fields (Maunsell and Van Essen, 1983). Concerning motion integration, Pack and Born (2001) have shown that the temporal dynamics of behavior can correspond with the firing rates of MT neurons. They showed that neuronal responses quickly progress from local to global motion direction in about 100 ms, suggesting that such integrative mechanisms are dynamical and progressive. These results pinpoint the key role of MT neurons in local motion analysis and global motion integration. Area MT and MST receive feed-back connections that may modulate the activity of their neurons (Salin and Bullier, 1995). However, these connections (mostly myelinated) introduce constant delays and are mostly related to higher level contextual modulations. Provided that motion extrapolation is implemented in one single cortical area, a finely structured set of diffusive mechanisms would be required. A potential candidate is naturally the dense network of lateral interactions found in sub-cortical and cortical structures involved in sensory processing and sensorimotor control. Of particular relevance is the role of the connectivity pattern in the emergence of a solution to this problem. In this paper we will focus on a smaller spatio-temporal scale and study the role of lateral, intra areal (mostly unmyelinated) connections.

A possible correlate of prediction may lay in the traveling waves of neural activity that may be observed on the cortical surface. Bringuier et al. (1999) was the first to show a precisely tuned synaptic integration field (Bringuier et al., 1999) [see (Sato et al., 2012) for a review]. Theoretical studies suggest that for such waves to exist, there should exist some specific anisotropy connectivity pattern (Bressloff and Coombes, 1998). It is established that the speed of propagation of activity along these mostly unmyelinated connections is of the order 0.1–0.4 m/s but there is an ongoing debate on their selectivity. In the primary visual cortex, a set of patchy connections in the long-range horizontal connections found in superficial layers of cortex (Bosking et al., 1997) that preferentially connect columns with similar orientation preference has been observed in ferrets. This is consistent with the fact that columnar interactions determine horizontal propagation of recurrent network activity in neocortex (Wester and Contreras, 2012). It has also been observed that activity in cat V1 spreads anisotropically for all orientation columns (Chavane et al., 2011). Anisotropies in the connectivity pattern necessarily lead to a wide range of traveling wave parameters (speed, direction) and introducing inhomogeneities can in addition lead to more complex wave profiles and possibly even wave propagation failure (Bressloff, 2001). However, the function of these traveling waves, and therefore the underlying structure of the intracortical connectivity, is mostly unknown.

### 1.3. Existing neuromorphic models for prediction

There have been numerous attempts at modeling generic predictive neural mechanisms. Here, we review some prototypical examples at different modeling levels, from a more abstract level to a neuromorphic implementation.

Following the idea of the Kalman filter as an adaptive predictive filter and extending the work of Montagnini et al. (2007), Bogadhi et al. (2011) proposed a hierarchical recurrent Bayesian framework to understand the behavioral response to motion extrapolation as observed in smooth pursuit eye movements. Indeed, probabilistic inference has been successful in explaining motion perception to a variety of stimuli (Weiss et al., 2002) under the hypothesis that sensory areas use predictive coding as a generic neural computation (Rao and Ballard, 1999). They are somewhat similar to engineering models proposed earlier (Nowlan and Sejnowski, 1995) but allow for a more explicit formulation of the underlying hypothesis. Such a framework accommodates uncertainty in the motion information of the measurement likelihoods (Weiss et al., 2002; Stocker and Simoncelli, 2006; Hedges et al., 2011). Representing uncertainty in the measurements and prior expectation gives a simple, yet powerful framework to investigate the predictive behavior of the system, and offers the possibility to optimally adapt to changes in the measurements. The approach from Bogadhi et al. (2011) allows for a mix of prediction and measurement based on their reliability measured from their respective variances. The combined estimate is used to drive the pursuit response. The hierarchical framework allows investigation of the adaptive behavior of pursuit as well as the role of prediction on motion integration as observed in pursuit responses. Such Bayesian models give a generic account of the motion extrapolation mechanism but do not provide a neural implementation.

A direct translation could in theory be performed by a probabilistic population code approach (Beck et al., 2008). This requires that neural responses represent probability distributions and assume “Poisson-like” spike response variability. Under that hypothesis, one could derive from a Bayesian model the architecture of a network of spiking neurons. Another approach is to use a global and generic functional cost for the problem (such as the free-energy of a system designed to track a dot) and derive the optimal system. Such endeavors allow one to propose a hierarchical neural architecture (Friston, 2009), which predicts behavioral results under visual occlusion for control and schizophrenic patients (Adams et al., 2012). Such models are in essence similar to other modeling approaches where neural activity is represented by average firing rate on a cortical map (forming a so-called neural field). Such models were successful in accounting for a large range of classical and non-classical receptive field properties of V1 including orientation tuning, spatial and temporal frequency tuning, cross-orientation suppression, surround suppression, and facilitation and inhibition by flankers and textured surrounds (Spratling, 2010). Similar models were applied to problems specific to motion detection and a link can be drawn between such solutions and classical solutions drawn in computer vision (Tlapale et al., 2010). However, these models do not take advantage of the specificity of computing with spiking neurons, that is the dual property of being able to integrate information and detect synchrony in the input.

Some models propose solutions related to motion extrapolation using neuronal networks (spiking and non-spiking). A recent model of spiking units (Lim and Choe, 2008) explains the phenomenon of the flash-lag effect (Nijhawan, 2008) by a motion-extrapolation mechanism provided by facilitating synapses, but acts on the single cell level only. Baldo and Caticha (2005) present a feed-forward network of leaky integrate-and-fire (LIF) neurons performing prediction, but that does not account for the role of recurrent connectivity abundant in cortical networks. In this regard, Liu and Wang (2008) proposed a more realistic recurrent network but focused on a binary decision task, whereas we aim at a more generic solution for the problem by studying prediction performance for a spectrum of possible directions. Recently, Jancke and Erlhagen (2010) used a recurrent neural field model to explain visual illusions like the Fröhlich, the flash-lag, and the representational momentum effects. Our approach is similar to theirs in the sense that the mechanism for motion-extrapolation can be seen in spreading activation to surrounding neuronal populations, but differs fundamentally in the way that connections are set up, as connection selectivity for directional tuning is not considered in their model.

As an intermediate observation, we see that though there exist a wide spectrum of models, a common feature is that these models use diffusive mechanisms implemented by the connectivity to propagate predictive information (probabilities, population activity, spikes) from a local to a global scale. The richness of behaviors is then mostly obtained by using different types of neurons (for instance by varying their polarity—excitatory or inhibitory, or the time constant of the synapses), which implements complex non-linear mechanisms such as gain control. This may be sufficient to account for motion extrapolation. However, it should also be highlighted that all these models assume a prediction in all directions and therefore that the connectivity is *a priori* isotropic. We challenge this assumption by introducing anisotropy in the connectivity as a key mechanism transporting motion information in a coherent manner.

### 1.4. Our approach: an anisotropic connectivity pattern implementing motion-based prediction

At the behavioral level, Yuille and Grzywacz (1989) have shown that motion integration in humans is highly dependent on the smoothness of the trajectory of the stimulus. Humans can detect a target dot moving in a smooth trajectory embedded in randomly moving dots, while the target dot is not distinguishable from noise in each frame separately. Introducing a preference for smooth trajectories, the activity from local motion detectors are made more coherent in space and time and this globally lowers the threshold for detecting stimuli moving in smooth versus segmented trajectories. In particular, during a transient blanking, it is most likely that such processes (along with the knowledge that the sensory input was indeed blanked and not definitively removed) underlie motion extrapolation. For instance, when a moving target disappears, smooth pursuit eye movements continue at the same velocity during the initial period of occlusion (Bennett and Barnes, 2003). Therefore, it seems that neural computations take advantage of the information about motion, but it is yet not clear how this can be done efficiently in a network of spiking neurons.

At an abstract level, a preference for temporal coherency of motion can be defined in a probabilistic framework. This was formulated theoretically by Burgi et al. (2000), who proposed a neural field implementation including local to short-range connectivity. However, it lacked the precision needed to efficiently represent realistic input sequences. In our earlier work (Perrinet and Masson, 2012), we implemented an efficient prior for smooth trajectories to investigate different aspects of spatio-temporal motion integration. Particularly, this model focused on the aperture problem and proposed that local, diffusive predictive coding is sufficient to infer global motion from local, ambiguous signals. The aperture problem is a challenging problem to study integration of local motion information (Pack and Born, 2001). The model proposed that instead of specific mechanisms such as line-ending detectors, the gradual spatio-temporal integration of motion relies on prediction based on the current knowledge of motion in terms of its velocity and position (motion-based prediction). Compared to previous models the main difference of this implementation is that, it is possible to predict that information about motion velocity at a known position will be transported in the direction given by the velocity.

Indeed in motion-based prediction, the retinotopic position of the velocity of motion is an essential piece of information that allows routing information and allow implementation of predictive coding on smooth trajectories. By including explicitly the dependence of local motion signals between neighboring times and positions knowing the current speed along a smooth trajectory, incoherent features should get canceled out, while coherent information should get progressively enhanced. As such, this context-dependent, anisotropic diffusion in the probabilistic representation of motion also results in the formation of a tracking behavior favoring temporally coherent features. Such a model was recently extended to account for motion extrapolation (Khoei et al., 2013) and has been able to replicate some behavioral results from Bogadhi et al. (2011). Our goal here is to show that the idea of motion-based prediction [as described in Perrinet and Masson (2012)] can be implemented in a generic network of spiking neurons through anisotropic connectivity and that this is sufficient to solve a motion extrapolation task. The novelty compared to previous studies is the transition from an abstract, probabilistic framework to a spiking neural network and the link between anisotropic connectivity to motion-extrapolation, a task of functional relevance. Of course, we will not exclude that other complementary solutions may exist, but we will argue that it constitutes one of the simplest solutions for a network of spiking neurons. For that purpose, we will use a classical implementation of recurrent networks using conductance-based integrate-and-fire neurons with three prototypical connectivities: random, isotropic or anisotropic. While the consequence of non-homogeneous connectivities has been somewhat explored (Voges and Perrinet, 2012), it is—up to our knowledge—the first study of the functional consequence of anisotropic connections in a large-scale neural network.

### 1.5. Objectives and outline

This paper is organized in the following order: First, we develop a network of spiking neurons with the connectivity directly drawn from the probabilistic modeling framework proposed for the solution to the aperture problem (Perrinet and Masson, 2012), and that was extended to the motion extrapolation problem (Khoei et al., 2013). We will include in Section 2.1 details on structure and implementation of the model but also details from the experimental and numerical aspects.

Then, we report results in Section 3 from simulations where we studied the network response to a disappearing moving dot under three different connectivities: random, isotropic or anisotropic.

Finally in the discussion (Section 4), we interpret these results in the light of current knowledge on probabilistic inference and dynamical systems, and we will discuss the limitations of the current study along with suggestions for future work.

## 2. Methods

### 2.1. Neuron parameters

Simulations were performed with PyNN (Davison et al., 2008) as interface to the NEST simulator (Gewaltig and Diesmann, 2007) on a Cray XE6 system using 96 cores. For analysis we used python modules numpy (Oliphant, 2007), scipy (Oliphant, 2007) and visualization was performed using matplotlib (Hunter, 2007). Neurons were simulated as LIF neurons with conductance based synapses. The membrane potential *V* of a neuron with index *k* obeys the following equation:

(1) $$C_{m}\frac{dV_{k}}{dt}=g_{l}\left(E_{l}-V_{k}(t)\right)+\sum_{j}[g_{j,k,E}(t)\left(E_{E}-V_{k}(t)\right)+g_{j,k,I}(t)\left(E_{I}-V_{k}(t)\right)],$$

where *j* is the index of the sources, $g_{j,k}(t)=w_{j,k}\cdot \exp\left(-\frac{t-t_{spike}}{\tau_{p}}\right)$ is the synaptic conductance time course with *p* ∈ {*E*, *I*}: τ_*p*_ are the synaptic time constants, and *E*_*p*_ is the reversal potential for excitatory (*p* = *E*) and inhibitory (*p* = *I*) synapses respectively, *g*_*l*_ is the constant leakage conductance, and *E*_*l*_ the leakage or resting potential. When the membrane potential *V* is above the threshold voltage *V*_thresh_ = −50 mV a spike is emitted and *V* is set to *V*_*reset*_ = −70 mV for a refractory period of τ_*refrac*_ = 1 ms. Table 1 lists the parameters used for both excitatory and inhibitory neurons. The cell and synapse parameters have been chosen to be in a similar range as seen in experimental studies [see Table 3 in the study by Rauch et al. (2003)] to allow for comparison between future modeling and experimental studies. The model is in principle not dependent on the cell parameters and different parameters would not change the fundamental outcome, but returning of other parameters like connection strengths would be necessary. The initial values of the membrane potentials are drawn from a normal distribution around *V*_*init*_ = −65 mV with a standard deviation of 10 mV.

**Table 1:** **Neuron parameters**.

Name	*C*_*m*_	*g*_*l*_	τ_*m*_	*E*_*l*_	*E*_*E*_	*E*_*I*_	τ_*E*_	τ_*I*_
Value	1	0.1	10	−70	0	−70	5	10
Unit	nF	μS	ms	mV	mV	mV	ms	ms

### 2.2. Tuning properties

The model is inspired by retinotopic cortical areas like areas V1 or V5/MT in primates. In our model, each neuron has four tuning properties: (*x*_*i*_, *y*_*i*_, *u*_*i*_, *v*_*i*_) parameterizing the center of the spatial receptive field of neuron *i* at position ${\vec{x}}_{i}=(x_{i},y_{i})$ and its preferred direction ${\vec{v}}_{i}=(u_{i},v_{i})$. The width of this receptive field defines the tuning selectivity of neurons and is parameterized by β_*X*_ and β_*V*_, respectively for space (*x* and *y*) and velocity (*u* and *v*). The spatial receptive fields are arranged in a hexagonal grid to optimally cover the input space which is set to span a 1 × 1 area in arbitrary units. As we will implement a network size of approximately 10^4^ neurons, this will in practice correspond to a spatial scale of the order of millimeters. Velocities should therefore be in the range of m s^−1^.

In order to have receptive fields for all possible directions (up to a certain maximum velocity of approximately $|{\vec{v}}_{max}|=4.0{\text{ms}}^{-1}$) at all positions, the midpoint of each of the 100 hexagonal grid cells contains neurons with ten different preferred velocities for ten different angles, hence 100 different preferred directions per hexagonal grid cell. The lengths of preferred directions are distributed according to a distribution favoring low velocities (Weiss et al., 2002) with a logarithmic scale for the speed according to Weber's law (Stocker and Simoncelli, 2006). In order to avoid boundary effects, both spatial dimensions are closed and continuous. This leads to a horn torus as input space, i.e., if a stimulus leaves the 1 × 1 space it reappears on the opposite side (so-called “pac-man topology”). This topology holds also for the network connectivity, e.g., connections reaching beyond the virtual border at *x*_*target*_ = 1 will be wrapped around. After all tuning properties are set, they get dispersed to account for natural variability (Paik and Ringach, 2011).

### 2.3. Input stimulus

A classical way of studying motion extrapolation is by presenting a moving target that travels behind an occluder for a short period of time. A seminal study used timing estimation by asking participants to make a button press response at the time they judge the occluded target to have reached a particular point (Rosenbaum, 1975). Motion extrapolation can be carried out for lateral motion with the target moving across the fronto-parallel plane, or for approaching motion, when the object moves toward the observer (DeLucia, 2004). Herein, we investigate visual, lateral motion extrapolation only. For simplicity, we study the network's response to a moving dot stimulus and the network's ability to predict the trajectory of the dot when it disappears behind an obstacle producing a blank gap in the input signal.

From the definition of the tuning properties of a neuron *i*, we may model the response to a moving dot as an inhomogeneous Poisson process with a parametrically defined envelope. Indeed, we will use the following input stimulus *L*_*i*_(*t*) as the envelope for a Poisson process with a maximum of 5 kHz (when *L*_*i*_(*t*) reaches 1) and a time step of 0.1 ms:

(2) $$L_{i}(t)=\exp\left(-\frac{\|{\vec{x}}_{\text{stim}}(t)-\vec{x_{i}}{\|}^{2}}{2\beta_{X}^{2}}-\frac{\|{\vec{v}}_{\text{stim}}-\vec{v_{i}}{\|}^{2}}{2\beta_{V}^{2}}\right)$$

where ${\vec{x}}_{i}$ is the neuron's receptive field central position, ${\vec{v}}_{i}$ the neuron's preferred direction, ${\vec{x}}_{stim}(t)$, (${\vec{v}}_{stim}$) is the position (direction) of the moving dot (see Figure 1). As the trajectory of the dot is rectilinear and constant, we have

(3) $${\vec{x}}_{\text{stim}}(t)={\vec{x}}_{\text{stim}}(0)+{\vec{v}}_{\text{stim}}\cdot t$$

The resulting inhomogeneous Poisson spike train is connected to the respective neurons via one excitatory synapse of strength *w*_*input*_ = 5 nS. This formalization allows to study for the different roles of theses parameters. In particular, the tuning width may play an important role as it is known that in low level visual areas (such as the retina), receptive fields are small (position is accurate, motion is imprecise) while in higher level areas motion is more finely represented, while position is less precise (as the receptive fields' size increase). In the rest, all neurons have the same tuning width defined by β_*X*_ = 0.15 and β_*V*_ = 0.15 *s*^−1^. The β_*X*, *V*_ values have been set so that a reasonable part of the network receives sufficient input from the moving dot stimulus. Increasing β_*X*, *V*_ would make the dot appear broader, whereas smaller β_*X*, *V*_ would make a smaller fraction of the network respond to the stimulus. Changes in the β_*X*, *V*_ parameters would not change the working concept of the model, but would require a retuning of connectivity parameters like number of connections and connection strengths.

For simplicity, we studied only networks in which excitatory neurons receive input because inhibitory neurons primarily provide a normalization mechanism in our model, even though this might not reflect real cortical circuits (Frégnac et al., 2003). All neurons receive additional noise in form of Poisson spike trains with a rate of *f*_*noise*_ = 2 kHz injected via excitatory and inhibitory synapses with a weight of *w*_*noise*_ = 4 nS to simulate the input from external networks. For all simulations of this paper, the network was stimulated with a dot moving at a speed of ${\vec{v}}_{stim}=0.5s^{-1}$ from left to right (and an initial position defined by ${\vec{x}}_{stim}\text{(t=0)}=(0.1,0.5)$). Crucially, during the blank phase, the stimulus vector in the network was permuted randomly at each time step among the whole excitatory population, so that the selectivity of the input was completely lost while keeping a similar input average frequency as compared to phases when stimulus is active. As the input vector is shuffled the network does not receive a coherent continuous input signal. During the blank phase, cells that are well-tuned to the stimulus receive input spike trains with a larger inter-spike-interval leading to a decrease in the effective input. This is due to the integration of post-synaptic potentials on the membrane in the context of LIF neurons. Compared to an empty input vector, this input vector shuffling during the blank phase elevates the mean membrane potential of the population slightly which can help the network to fill the blank phases with meaningful input.

### 2.4. Network connectivity

The network study consists of an excitatory population with *N*_*exc*_ = 13000 neurons and an inhibitory population with *N*_*inh*_ = 2520 neurons (that is, with a ratio of 16.2% inhibitory cells over the whole population). Both populations are mutually and recurrently connected in one of the following ways which will be explained in the next sections: randomly, isotropically or anisotropically.

#### 2.4.1. Random and isotropic connectivities

Connections within and between populations can be set up in an isotropic manner that does not depend on the source or target neuron's tuning properties. When neurons are connected in this way, connection probabilities are computed according to:

(4) $$p_{ij}=p_{\text{max}}\cdot \exp\left(-\frac{d_{ij}^{2}}{2\cdot \sigma_{X}^{2}}\right)$$

where *p*_*max*_ is a normalizing factor and $d_{ij}=\left\|{\vec{x}}_{i}-\vec{x_{j}}\right\|$ represents the distance (in visual space) between both neurons. *p*_*max*_ is set so that the total number of connections between two populations drawn isotropically is equal to an overall probability of *p*_*k*, *l*_, (*k*, *l*) ∈ {*E*, *I*}. The connection probabilities utilized are: *p*_*EE*_ = 0.5%, *p*_*EI*_ = 2%, *p*_*IE*_ = 2%, *p*_*II*_ = 1%.

Weights are drawn from a normal distribution with mean μ^*iso*^_*w*_ and standard deviation σ^*iso*^_*w*_ = 0.2 · μ^*iso*^_*w*_. The value of μ^*iso*^_*w*_ is set so that the expected sum of incoming weights equals a certain target value *w*_*kl*_ specific to the type of the source and target population (*k*, *l* ∈ {*E*, *I*}): *w*_*EE*_ = 0.3 μS, *w*_*EI*_ = 1.8 μS, *w*_*IE*_ = 0.8 μS, *w*_*II*_ = 0.15 μS (if not stated differently).

Delays are drawn from a normal distribution with a mean value μ^*iso*^_δ_ = 3 ms and a standard deviation of σ^*iso*^_δ_ = 1 ms. Self-connections have been discarded. A completely random connectivity may be then achieved by setting σ_*X*_ to a sufficiently big value (relative to the scale of the spatial period). This results in a flat, uniform probability of connection over the whole population.

#### 2.4.2. Anisotropic, motion-based prediction connectivity

Inspired by motion-based prediction (Perrinet and Masson, 2012), we may define a connectivity by wiring neurons that are linked by a smooth trajectory with a higher probability. Connectivity will then be specifically anisotropic as it provides a mechanism for motion-based prediction by diffusing motion information across the network in a forward, asymmetric manner. Specifically, we will take advantage of the latency that exists between neurons in the same cortical area and use that parameter to connect cells matching a smooth trajectory. The motivation underlying this formula is based on the idea that smooth trajectories are more likely seen in natural scenes and are promoted by the network connectivity. If the target position is situated at the position where the source neuron predicts the stimulus to be in a certain time τ_*ij*_ and if the target neuron predicts the stimulus to move in a similar direction ${\vec{v}}_{j}$ as the preferred direction of the source neuron ${\vec{v}}_{i}$, the source neuron connects with a high probability to the target neuron.

As a consequence, the connection probability is computed from the tuning properties of the source neuron *i* and target neuron *j* according to the sampling of the prior defined in Perrinet and Masson (2012):

(5) $$p_{i,j}=p_{\text{max}}\cdot \exp\left(-\frac{{\left\|{\vec{x}}_{i,\;j}^{*}-\vec{x_{j}}\right\|}^{2}}{2\cdot \sigma_{X}^{2}}\right)\cdot \exp\left(-\frac{{\left\|{\vec{v}}_{i}-{\vec{v}}_{j}\right\|}^{2}}{2\cdot \sigma_{V}^{2}}\right)$$

(6) $${\vec{x}}_{i,\;j}^{*}={\vec{x}}_{i}+{\vec{v}}_{i}\cdot \tau_{i,j}$$

(7) $$\tau_{ij}=\frac{\left\|\vec{x_{i}}-\vec{x_{j}}\right\|}{\left\|{\vec{v}}_{i}\right\|}$$

**Figure 2:**
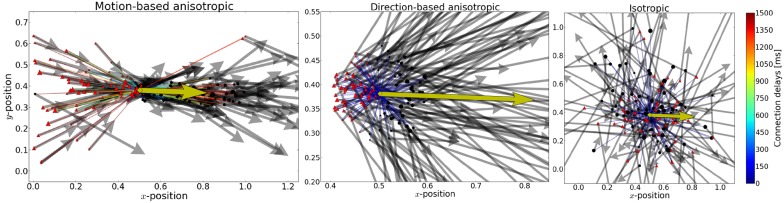
**From aniso- to iso-tropic connectivities**. We propose that the local connectivity pattern of lateral connections play a crucial role in solving the motion-extrapolation problem. In order to show that, we compared different connectivity patterns in response to the blanked input (see Figure 1). In the three panels, we show the incoming and outgoing connections for the same single neuron (as marked by the yellow diamond) for different connection rules. The preferred direction of that neuron is shown by the yellow arrow. Cells targeted by the yellow cell are marked with black circles and cells projecting to the yellow cell are marked with red triangles. The relative weights of incoming (outgoing) connections are indicated by the size of the source (target) neuron, respectively. The preferred direction of source and target neurons is shown by solid arrows. Connection delays are color coded. **Left:** Motion-based prediction anisotropic connectivity. Inspired by previous work on motion-based prediction (Perrinet and Masson, 2012), we propose a first pattern of connectivity based on connecting a source neuron to a target neuron if and only if the position and velocity of the target is compatible with a smooth trajectory that would originate from the source neuron. The strength of this prediction is parameterized by the width of the lateral tuning selectivity and here we show the prototypical pattern for σ_*X*_ = 0.3 and σ_*V*_ = 0.3 ms^−1^ (as used for the simulations). **Middle:** Direction-dependent connectivity. To create a more realistic connectivity pattern, we used the same rule but independently of speed (i.e., the modulus of velocity), but only as a function of the direction of motion. The target neuron is connected if and only if its direction is close to the source's direction and if its position is predicted to be in the direction given by the source neuron. Additionally, to account for physiological constraints on lateral interaction, only connections within a radius of *r*_*Conn*_ = 0.10 or latencies shorter than 100 ms are allowed. This leads to a more local connectivity and smaller connection delays compared to the previous connectivity. We show here the resulting connectivity pattern for σ_*X*_ = 0.3 and σ_*V*_ = 0.3 ms^−1^ with the motion-dependent connectivity and σ_*X*_ = 1.0 and σ_*V*_ = 1.0 ms^−1^ with the direction-dependent connectivity. **Right:** An isotropic connectivity pattern was chosen as a control. There is no prediction in velocity space, but we still predict that activity should diffuse locally, as the connection probability drops with the distance between cells.

In this formulation, ${\vec{x}}_{i,\;j}^{*}$ is the position predicted for a motion that would leave the source neuron's receptive field (therefore from position ${\vec{x}}_{i}$ and with velocity ${\vec{v}}_{i}$) after a latency τ_*ij*_. Then, parameter τ_*ij*_ corresponds to the expected latency knowing the respective position and velocity of source and target neurons. In Equation (5), the parameters σ_*X*_ and σ_*V*_ determine the strength of the tuning properties of motion-based prediction. Unless stated otherwise, we will use σ_*X*_ = 0.1 and σ_*V*_ = 0.1^−1^ (see Figure 2). Note that the precision of prediction in the velocity domain (that is σ_*V*_) determines a scaling factor for the degree of anisotropy: the lower σ_*V*_ is, the more the outgoing connections of a neuron are aligned with the preferred direction of the source neuron. Note also that only σ_*V*_ includes the predictive prior on velocity and that we may retrieve an isotropic connectivity by setting σ_*V*_ to a sufficiently high value.

The probabilities are then sorted and each target neuron receives input from 0.5% of the source neurons that have the highest connection probability. Those 0.5% highest probabilities are converted to connection weights so that the sum of incoming weights per neuron equals a certain target value *w*_*kl*_ specific to the type of the source and target population (*k*, *l* ∈ {*E*, *I*}): *w*_*EE*_ = 0.20 μS (for motion-based connectivity and *w*_*EE*_ = 0.25 μS for direction-based connectivity), *w*_*EI*_ = 1.8 μS, *w*_*IE*_ = 0.8 μS, *w*_*II*_ = 0.15 μS (these values are only for the example networks and might differ depending on the exact implementation and require an adjustment for different network sizes). This means the connectivity becomes deterministic (based on the tuning properties of the source and target cell) and the term probability refers only to the overall selection of source cells in the network.

#### 2.4.3. Anisotropic, direction-based prediction connectivity

However, if we use the previous equation to connect cells [Equation (5)], and scale our network realistically, it appears that latencies depend on the velocity coded by the cells, and in turn, this leads to unrealistically high delay values with the range of velocities we used. As a consequence, we defined another way of setting up the connectivity which only take into account the angle between source and target cells and the angle between the directions coded for by the source and target cells. It is therefore independent of the preferred speed (modulus of velocity) of the neurons and on the latency used to connect the cells.

We use von Mises probability distribution functions to define the tuning in the range of all directions:

(8) $$p_{i,j}=p_{\text{max}}\cdot \exp\left(\frac{\cos\left({\vec{x}}_{j}-\vec{x_{i}},\vec{v_{i}}\right)}{\sigma_{X}^{2}}\right)\cdot \exp\left(\frac{\cos\left({\vec{v}}_{i},{\vec{v}}_{j}\right)}{\sigma_{V}^{2}}\right)$$

The first term guarantees that information spreads in the direction that is preferred by the source cell (and where σ_*X*_ gives approximately the width of tuning in radians). The second term ensures that information is passed only to cells that code for motion moving in a similar direction as preferred by the source cell (and where similarly σ_*V*_ gives approximately the tuning width in radians). Note that in position-velocity space, the probability of connection is maximal in the direction given by the preferred velocity of the source cell and centered on the position of that cell's receptive field. The density therefore defines a cone around this half-line, defined by widths σ_*X*_ and σ_*V*_ [see middle panel in Figure 2]. Note that this formulation may be derived from the formulation of motion-based prediction by lowering the strength of prediction on the radial component of velocity. As such, this connection probability gives a similar mechanism for promoting smooth trajectory, and provides the diffusion of motion information in the direction detected by the network. A comparison of these two network connectivities is visualized in Figure 2.

Whereas encoding and decoding of direction information is now largely understood in various neuronal systems, how the human brain accurately represents speed information remains largely unknown. Speed tuned neurons have been identified in several early cortical visual areas in monkeys. However, how such speed tuning emerges is not yet understood. A working hypothesis is that speed tuned neurons non-linearly combine motion information extracted at different spatial and temporal scales, taking advantage of the statistical spatiotemporal properties of natural scenes. Furthermore, the population code underlying perceived speed is not yet elucidated and therefore we are still far from understanding how speed information is decoded to drive and control motor responses or perceptual judgments. As a consequence, such a connectivity profile will serve as a further control to test if restraining the prediction to direction is sufficient to solve the motion extrapolation problem.

### 2.5. Choice of parameters

The number of receptive fields has been set so that the four-dimensional space of tuning properties is covered with a reasonable density of cells. Decreasing the number of receptive fields would decrease the number of cells in the network and would impede the diffusion of information between cells. This is because the weight of connections is sensitive to the distribution of cells in the tuning property space, and a over-sparsely populated tuning property space can lead to unwanted effects for activity spread in the network. The parameters describing receptive field sizes, β_*X*_ and β_*V*_, determine the distribution of the input signal in the network. They have been chosen so that a small part of the network receives a sufficient amount of excitation that brings this small part above the spike threshold and initiates the spread of activity, and by that, the diffusion of motion-information in the network. Increasing β_*X*, *V*_ would make the stimulus appear fuzzier and, consequently the extrapolation task more difficult. A decrease of β_*X*, *V*_ would make the stimulus appear sharper. But it would not necessarily make the task easier since the source of activity would be smaller and the seed for the diffusion of information could possibly be too small to propagate through the network, depending on the network connectivity parameters. The parameters determining the network connectivity *p*_*k*, *l*_ and *w*_*k*, *l*_, (*k*, *l*) ∈ {*E*, *I*} were chosen to be in a range comparable to physiological values for large networks. Especially the connection weights needed to be fine tuned to solve the motion-extrapolation task. Redistributing the tuning properties could easily lead to instabilities, i.e., that the trajectory could not be extrapolated, and too high weights could lead to an explosion of activity in the network.

### 2.6. Prediction readout

A crucial issue when trying to map a Bayesian inference algorithm to a network of spiking neurons is to understand how probability can be expressed in terms of neural activity. Herein, we applied a simple vector averaging method to infer the prediction about stimulus position and direction from the activity of the excitatory population. Indeed such decoding scheme may be justified as a simple implementation of probabilistic codes as done by Beck et al. (2008). Their approach requires several assumptions which are not guaranteed in our model: First of all, neurons are assumed to have Poisson-like spiking statistics, which is obviously not true in our model since activity is strongly driven by the stimulus and hence neuronal activity is not Poisson-like (see 3). Secondly, they assume that network activity is uncorrelated on timescales of 50 ms, which is likewise not realistic for our model. Furthermore, their approach works on probability distributions gained over several trials, which could principally be done with our model, but it is computationally more expensive than the single-trial vector-averaging method described above. However, this provided a decoding approach which seemed to robustly represent the activity in the network.

In particular, we used a similar formulation as the decoding framework proposed for neurons in area MT (Jazayeri and Movshon, 2006). Indeed, the definition of our model fits well to their implementation. In both models, the activity of sensory neurons is pooled in a simple additive feed-forward architecture, In contrast to their model, we extend the application beyond the angle of motion and apply the readout framework to position and direction. More precisely, the tuning properties are in the exponential family and tile uniformly the position-velocity space. Thanks to the definition of the tuning selectivity of the neurons in the network, the position and velocity corresponding to the Maximum Likelihood estimation corresponds to the average over all neurons of each central tuning parameter weighted by the activity of the neuron (independently of β_*X*_ and β_*V*_ as they are uniform for all neurons). To define a continuous activity at each time bin a weight *p*_*i*_(*t*) is defined for each excitatory neuron *i* based on the number of output spikes fired during a time bin *t*:

(9) $$p_{i}=n_{i}(t)/\sum_{i}^{N_{\text{exc}}}n_{i}(t)$$

where *n*_*i*_(*t*) is the number of spikes fired by neuron *i*. The time bin size was set to 50 ms, but it could be chosen differently without qualitative changes.

Such decoding schemes are classically implemented on unbounded variables. However, we defined space on a torus in order to avoid edge effects. Hence, the network average must be computed for circular quantities (Mardia and Jupp, 2009): The idea behind Equation 10 is that in order to compute the mean of a circular quantity, the position and direction first need to be transformed into an angle, which is then projected to the 2D unit circle where the arithmetic mean is computed. After that, the angle that the mean position forms is transformed back from an angle to space. For positions, this takes the form:

(10) $$x_{\text{pred}}^{\text{net}}(t)=1+\frac{1}{2\pi }\text{arctan2}(\sum_{i}^{N_{\text{exc}}}p_{i}(t)\sin\left(2\pi x_{i}-\pi \right),\sum_{i}^{N_{\text{exc}}}p_{i}(t)\cos\left(2\pi x_{i}-\pi \right))$$

where *x*_*i*_ is the center of the spatial receptive field of neuron *i* (the same formula is applied to compute *y*_*pred*_(*t*)). The subtraction of *pi* in the sin and cos functions is necessary to map the interval of position which is between 0 and 1 to the interval of (−π, π) required for the projection of position on the unit circle.

Similarly, for reading our the direction of the stimulus predicted by the network:

(11) $$v_{\text{pred}}^{\text{net}}(t)=\frac{1}{\pi }\text{arctan2}\left(\sum_{i}^{N_{\text{exc}}}p_{i}(t)\sin\left(\pi v_{i}\right),\sum_{i}^{N_{\text{exc}}}p_{i}(t)\cos\left(\pi v_{i}\right)\right)$$

The difference between Equations 10 and 11 lies in the fact that positions are bound to be between 0 and 1, whereas directions can be negative and larger than 1 or −1, which changes the transformation to and from angles for position and direction. Taken together, this gives an easy decoding scheme from the neural activity to a probabilistic read-out.

## 3. Results

### 3.1. Anisotropic diffusion transports information during the blank

Recurrent excitatory connectivity is a candidate for providing mechanisms for motion-based prediction. In order to prove the functionality of our approach, we show results for single example networks of three different connectivity types applied to the recurrent excitatory population: isotropic, anisotropic motion-based (speed tuning dependent) and anisotropic direction-based (speed tuning independent). For simplicity, the other connectivities are set up according to the isotropic scheme. Networks using the anisotropic scheme for one or more of the other pathways (*E* − *I*, *I* − *E*, *I* − *I*) could be tuned to perform similarly (not shown).

The connectivity in our model is mainly controlled by two parameters: the sum of incoming weights and the number of connections received by a cell. The sum of incoming weights for excitatory-excitatory connections have been tuned so that the activity initiated by the stimulus is strong enough to propagate through the network when the stimulus is turned off after 400 ms of input driven activation. Weights from the excitatory to the inhibitory population have been chosen so that inhibitory neurons exhibit a reasonable level of average activity of approximately 5 Hz when the stimulus is driving the excitatory population. The role of inhibitory to excitatory connections is to balance the network activity when the stimulus is active after the blank. In contrast to balanced random networks, the inhibitory to excitatory weights could not be set to high values that compensate for the higher number of excitatory neurons as in previous models [e.g., (Brunel, 2000; Morrison et al., 2007)] because strong isotropic inhibitory feedback would silence excitatory neurons in the vicinity and impede the propagation of motion information during the blanking period. The interplay between the excitatory and inhibitory populations is crucial for balancing network activity, but more importantly, for suppressing activity that creates false predictions about the target trajectory. All connectivity parameters were tuned so that the spread of activity within the excitatory population is strong enough to fill a realistically long blanking period, where the average duration of a single blink is between 100 and 400 ms (Schiffman, 2001).

**Figure 3:**
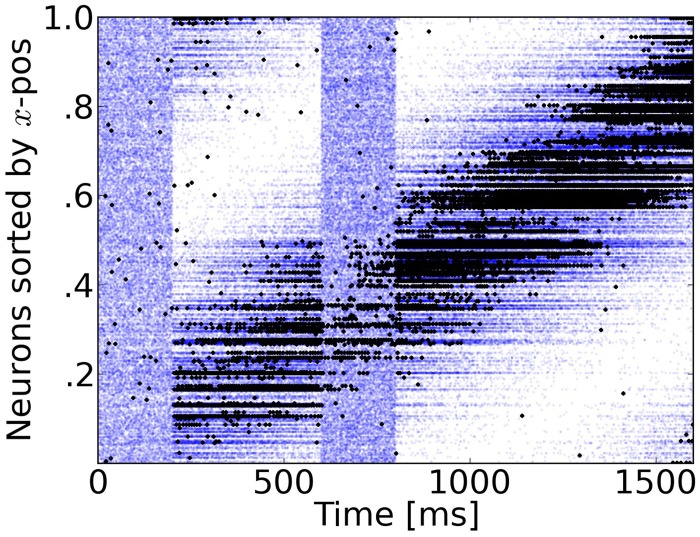
**Rasterplot of input and output spikes**. The raster plot from excitatory neurons is ordered according to their position. Each input spike is a blue dot and each output spike is a black dot. While input is scattered during blanking periods (Figure 1), the network output shows shows some tuned activity during the blank (compare with the activity before visual stimulation). To decode such patterns of activity we used a maximum-likelihood estimation technique based on the tuning curve of the neurons.

Before stimulus onset, the network input consists of background noise that persistently drives the network at low firing rates. The stimulus spike trains are dispersed over the whole excitatory population (see Figure 3). As this input is not coherent, the type of connectivity has no effect on the activity before onset. The stimulus activates the network between 200 and 600 ms of the simulation before another blank phase of 200 ms in which the input is equal to the phase before stimulus onset. Neurons that are well tuned to the stimulus fire at very high rates (temporarily up to 250 Hz) when the stimulus is present. This is due to the strong input stimulus and the amplification by the recurrent excitation. The average firing rate of neurons being active at least once during the simulation grows to up to 20 Hz when the stimulus is persistently present.

**Figure 4:**
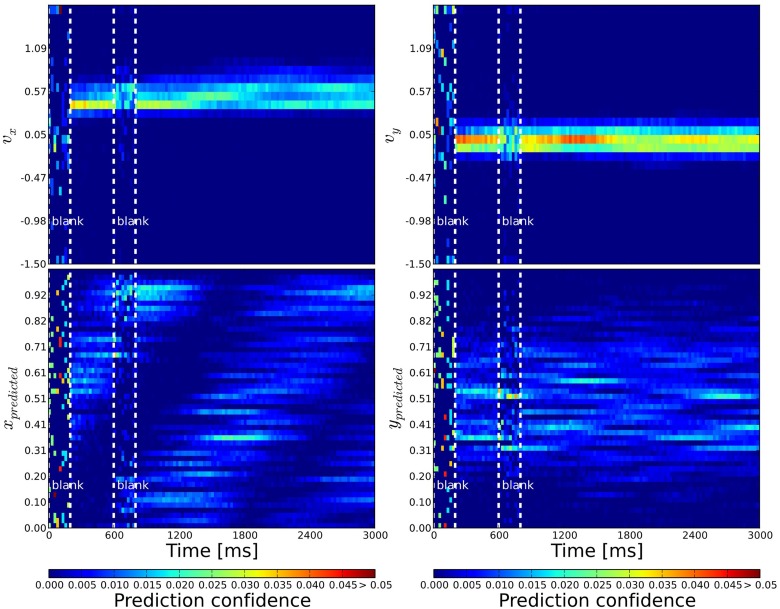
**Probabilistic population decoding and the resolution of the motion extrapolation problem using anisotropic connectivity**. The computed prediction confidence resulting from our simulations is shown using the motion-based anisotropic connectivity pattern with respect to time. The three vertical dashed lines correspond to: the onset of the stimulus, the onset of the blank and finally the reappearance of the stimulus, respectively. The cells' prediction confidence is defined in Equation 9 and have been sorted and binned according to their tuning properties. The accumulated confidence within each time bin is color coded. **Top**: left (right): We show the prediction confidence of movement direction *u* = *v*_*x*_ (*v* = *v*_*y*_). **Bottom**: left (right): Prediction confidence about *x* and *y* position, respectively. While information is distributed before stimulation and quickly converges during stimulation, it is predicted during the blank: the motion extrapolation is solved and information is very quickly recovered at the reappearance of the stimulus.

During the blank phase the global network activity drops rapidly to a low average rate of approximately 2 Hz and those neurons that convey the remaining motion information fire approximately 5–15 spikes during the blanking period, as individual output rates remain elevated to levels of 25–75 Hz. Due to the anisotropic connectivity the activity triggered by the stimulus propagates through the network in the direction that was initiated by the target (see Figures 3, 4).

We observed that there needs to be a balance between stimulus induced excitation and the recurrent excitation: When recurrent excitation is too strong, the internal neural dynamics dominate over the activity triggered by the stimulus and it is likely that false tracking behavior occurs, i.e., network activity spreads too fast in the direction of the stimulus and the predicted trajectory gets ahead of the target. When recurrent excitation is not strong enough, the network activity fails to fill the blank by its own dynamics like in the network with isotropic connectivity.

The connectivity parameters σ_*X*_ and σ_*V*_ need to be chosen differently for the two anisotropic networks, because their role in determining the connection probability between cells is slightly different according to Equations 5 and 8. For motion-based (*MB*) connectivity we used σ_*X*_, *V*^*MB*^ = 1, and for direction-based (*DB*) σ_*X*_, *V*^*DB*^ = 0.5. When σ_*X*_ < σ_*V*_ two main effects could be observed. As the network connectivity is more specific in the spatial domain, the prediction performance in the target direction tends to be lower and in the target position gets more precise. But the prediction can get ahead of the stimulus because excitation spreads to fast along the predicted trajectory. In the opposite case, when σ_*V*_ < σ_*X*_, the prediction of target direction gets more precise. Since the connectivity is spatially more distributed, the network is less likely to fill the blank because excitation is diluted across space.

### 3.2. Reading-out the population response

Jazayeri and Movshon (2006) presented a framework for a generic representation of likelihoods of sensory stimuli by neural activity. Here, we used a similar approach (see 2.6) which allows us to transform the binary spiking activity into a continuous valued representation of probability about the target motion. By these means, the activity of individual neurons can be interpreted as time-continuous confidence measure.

The phase before stimulus onset is like a prior probability. It is dominated by noisy activity that seems uniform and does not converge into a coherent probability distribution (see Figure 4). At stimulus onset, the network activity increases instantaneously and the probability distribution changes into a meaningful representation of motion information. During the blank period, the network activity drops rapidly (sometimes more gradually) and the probability distribution becomes more noisy, but changes less dramatically. Hence, despite the overall decrease in activity, information is not lost when the stimulus disappears. Instead, activity continues to propagate through the network, driven by the anisotropic connectivity. When the stimulus reappears the network activity grows again and continues to grow up to an average rate of 20 Hz until it is counterbalanced by the inhibitory feedback.

### 3.3. Motion-based predictive anisotropic diffusion solves motion extrapolation

In order to get a global estimation of the motion information we combine the probability estimates of individual neurons as described earlier (see 2.6) by a linear weighting of their time-varying activity. This provides a single valued, time-continuous prediction, i.e., readout signal of target position and direction. We will now compare the maximum confidence response for the three different connectivities to the exact same input in order to investigate the effect of the network connectivity on the readout signal.

**Figure 5:**
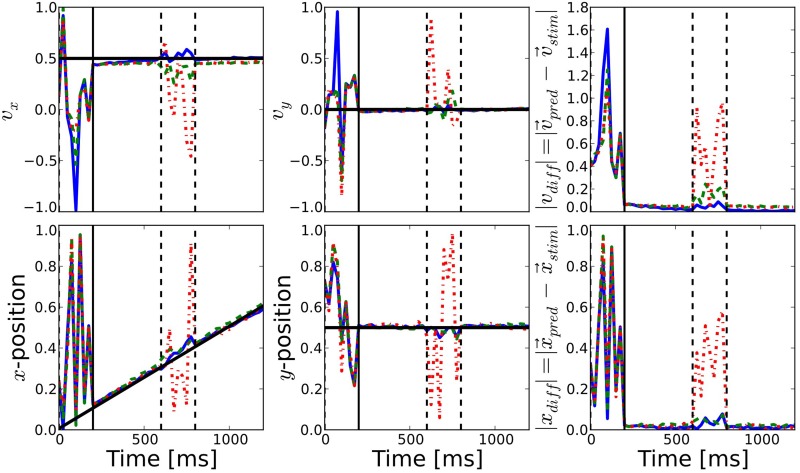
**Comparison of prediction performance for the different connectivities**. The performance of direction (**top**) and position (**bottom**) prediction as decoded from the network activity is shown (see Equations 10, 11). First and second columns show the horizontal and vertical components, respectively, while the last column shows the mean squared error of the predicted position with respect to the known position of the target. The color of the lines correspond to the different connectivities presented in Figure 2: motion-based prediction (solid blue), direction-dependent prediction (dashed green), isotropic (dash-dotted red). While an isotropic connectivity clearly fails to predict the fate of the input during the blank, we show here that the anisotropic connectivities may efficiently solve the motion extrapolation problem, even with an approximate solution such as the direction-based prediction.

Before stimulus onset the readout signal of all three networks follows the same noisy time course (see Figure 5). After stimulus onset and after the blank, all three estimations coincide with the actual target position and are very close to the target direction. This shows that our simple linear decoding approach is sufficient to translate the network activity into a meaningful readout signal.

The difference between the three networks can be seen during the blank phase. During this period, the readout signal of the network with the isotropic connectivity returns to the noisy time course, just like before the stimulus onset. In contrast, readout from the networks with anisotropic connectivity continues to give a precise estimation of the target position and direction of motion, as can be seen from the low prediction error (see the right most columns of Figure 5). The readout from the direction-based connectivity is less accurate than the motion-based connectivity, but it still shows that the direction-based diffusion mechanism allows for inference of the target position and direction during the blank phase. Thus, it can solve the motion extrapolation problem.

In some experiments, we observed that direction prediction appears to be biased toward higher velocities - especially during the blank. Improvements to the connectivity rule might be necessary to gain a “perfect” prediction performance (zero root-mean-square error). The reason for the drift toward higher velocities can be seen as an unbalanced distribution of incoming weights. Neurons with higher velocities are more seldom and hence have less cells with similar tuning properties in their vicinity. Due to the fact that all cells receive the same sum of incoming weights, the comparatively few cells that project to cells with high preferred velocities do this via few, strong connections, possibly leading to the observed drift and instabilities in the network dynamics. This could possibly be solved by improving how probabilities are mapped to connection weights, e.g., by introducing a non-linearity that prohibits weights above a certain value. Nevertheless, it was not our objective to present an optimal ad-hoc connection algorithm that gives perfect prediction performance, but to prove the fact that anisotropic, tuning property-based connectivity could be an important mechanism achieving motion-based prediction.

### 3.4. Conclusion

The comparison of the prediction performance of the three different networks shows two main points. Anisotropic connectivity provides a mechanism for the diffusion of motion information, which is relevant to predict future trajectories in noisy environments where the flow of information is interrupted frequently. Also, our simple approach to read out network activity linearly is sufficient to solve the given task, and does not require knowledge about probability distributions gained over many trials.

## 4. Discussion

### 4.1. Summary and comments

Following our previous study (Perrinet and Masson, 2012), we have confirmed that anisotropic diffusion of information is a sufficient mechanism to realize motion-based prediction as tested by the moving-dot blanking experiment. We have studied the role of different anisotropic and isotropic connectivity patterns and have shown that network connectivities that take into account the tuning properties of neurons and prefer smooth trajectories are more efficient in predicting the trajectory of a disappearing moving stimulus than isotropic networks. The main contribution of this study is to show that anisotropic diffusion of motion information can be implemented in networks of spiking neurons and thus could be a generic mechanism for motion-based prediction. Furthermore we have presented, to the best of our knowledge, the first model for motion prediction using spiking neurons and selective anisotropic connectivity that is inspired by a probabilistic framework.

The presented model is certainly limited and unrealistic in many ways. We have intentionally chosen a simple model (in terms of neuronal and synaptic features) to focus on the effects of connectivity patterns and to explore possible future applications for neuromorphic hardware systems (Schemmel et al., 2010; Brüderle et al., 2011). One of the main limitations consists of using long interconnection delays that are necessary to achieve its main function in the current state of the model. According to the spatial scale and the conduction delays of cortical networks, the connection delays resulting from our model are not on the same order of magnitude. Assuming that the concept of anisotropic diffusion of information operates in motion processing networks, neuronal mechanisms other than such long delays would be required to achieve the desired predictive function. One reason for long delays is likely the size of our network and that we are sub-sampling neurons in comparison to realistic cortical network sizes. In larger networks it would be sufficient to have recurrent excitation working on a more local scale and long-range connectivity would implement the transport of an expectation signal, possibly in the subthreshold domain. We have successfully explored one possibility to relieve the need for long delays by constraining the connectivity to more local scales, which reduced the required delays by more than one order of magnitude. Instead of the axonal delays as employed by our model, dendritic delays, long synaptic time constants (provided e.g., NMDA or GABA-B currents), or a combination of those three mechanisms could be used to implement the same principle. In summary, larger network sizes and longer synaptic time constants would likely help to realize our approach with shorter, more realistic connection delays. The fact that both models with longer and shorter delays show very similar performance could be a hint for the generality of the idea of motion information being transported by anisotropic connectivity.

### 4.2. Context of existing models

Based on early ideas by Hubel and Wiesel (1962), models employing anisotropic connectivity have been used to describe orientation selectivity (Finette et al., 1978) and its general use for visual information processing of static images with non-spiking units (Rybak et al., 1991). Models that are more similar to ours in motion coherence and the representation of motion trajectories [e.g., (Burgi et al., 2000; Jancke and Erlhagen, 2010)] do not use anisotropy in setting up the network connectivity. Other continuous recurrent network models have been used for various tasks like spatial working memory (Compte et al., 2000) and categorical discrimination with veridical judgment of motion (Liu and Wang, 2008). Our model works on a different level, but combines the functional features of previous models in the sense that motion trajectories are represented in a spiking and probabilistic way. Prediction signals are transported through recurrent connectivity, but none of the earlier models has shown that anisotropy could be a key element for this.

The dynamics of our network show some similarity to synfire chains (Prut et al., 1998) and we believe a model with similar dynamics and functionality could be implemented by attractor networks making use of a columnar organization (Lundqvist et al., 2006) that is prominent in motion processing areas like are V5/MT (Albright et al., 1984). Work by Bressloff (2001) has shown that weak heterogeneities in excitable neural media can lead to wave propagation failure. We have shown that in principle, it is favorable to have heterogeneity (i.e., the anisotropy in connectivity) to promote the spread of activity in a meaningful way. Still, there is much experimental evidence showing that cortical networks can spread activity in the form of traveling waves (Sato et al., 2012), and it is believed that long-range horizontal connections might be one of the underlying mechanism. It is arguable how well the conclusions of the Wilson-Cowan formalism used by Rybak et al. (1991); Bressloff (2001); Jancke and Erlhagen (2010) can be translated into the context of spiking networks, especially if the neuronal and synaptic machinery get more complex. We leave it for future analysis to determine if our network model could show behaviors similar to ones observed in experiments [for a review on traveling waves see Sato et al. (2012)].

We applied a simple vector averaging strategy to decode the position and motion direction from the network's response (Georgopoulos et al., 1986), but the optimal way of decoding is up to debate. Several studies (Priebe and Lisberger, 2004; Pack et al., 2005) suggest a vector averaging approach with a bias term to estimate speed, but more recent experiments suggest that “perceived speed is not based on a labeled-line interpretation of MT cells” (Krekelberg et al., 2006). Alternative decoding approaches involve a winner-take-all mechanism (Liu and Wang, 2008) or probabilistic codes (Beck et al., 2008). We may use an existing method to decode the optimal estimate from the population of neurons as is done in real neural data (Jazayeri and Movshon, 2006), though our goal here was to show in a simple way that anisotropic and selective connectivity could be of great importance for motion prediction.

Based on the results of our model, we predict that the connectivity in higher cortical motion-processing areas like MT or MST is not isotropic, but that effective connectivity between cells depends on their tuning properties. A sign of this anisotropic, tuning-property based connectivity could possibly be seen in future experiments similar to those in Guo et al. (2007) in the form of an anticipatory signal in cells that “expect” to receive stimulus input via the recurrent network connections.

### 4.3. Outlook and future work

After having shown a proof-of-concept for the idea that motion-based prediction can be achieved through anisotropic connectivity, many problems could be explored by the presented framework. One of the most urgent challenges in our view is the question how the recurrent connectivity can develop in a self-organized and robust manner. In order to integrate our model into the visual hierarchy, we need to understand how the tuning properties introduced in our model could be constructed through either feed-forward connections from lower cortical areas, through recurrent mechanisms that shape the desired properties, or both. Similarly important is the question of how a selective connectivity involving the inhibitory population influences the effective receptive field sizes along with the performance and stability of the presented framework. Another future challenge future is to use our probabilistic framework of spiking neurons for more realistic input and toward real-world applications.

### Conflict of interest statement

The authors declare that the research was conducted in the absence of any commercial or financial relationships that could be construed as a potential conflict of interest.
